# Application of Fly Ash Derived Zeolites in Warm-Mix Asphalt Technology

**DOI:** 10.3390/ma11091542

**Published:** 2018-08-27

**Authors:** Agnieszka Woszuk

**Affiliations:** Department of Roads and Bridges, Faculty of Civil Engineering and Architecture, Lublin University of Technology, Nadbystrzycka 40, 20-618 Lublin, Poland; a.woszuk@pollub.pl; Tel.: +48-815-384-375

**Keywords:** Warm-Mix Asphalt, synthetic zeolite, dynamic viscosity

## Abstract

In recent years, numerous studies have been carried out on new technologies allowing to reduce of mix asphalt production temperatures. One of the possibilities is to foam the asphalt with “water-containing” additives, which include zeolites. So far, mainly synthetic zeolites of the Linde A structure type, obtained from chemical reagents, and natural clinoptilolite have been used in WMA technology. In this studies, the synthetic zeolites produced from fly ashes with 4 different types of crystalline structure were analyzed. Zeolite materials were characterized by textural parameters and thermal analysis. The amount of zeolite added to asphalt was 0, 3, 5, 7 wt % in relation to the weight of asphalt. Determination of dynamic viscosity was performed at two temperatures: 135 and 160 and 4 time intervals. The tests were performed for two asphalt binders: 35/50 and 100/150 penetration grade. As a conclusion, it was found that the viscosity of asphalt pastes with zeolitic materials increases with the increase in the amount of zeolite added. The increase level depends mainly on the textural parameters. The potential usefulness of fly ash derived zeolites in the process of asphalt foaming, which depends mainly on the amount of water contained in the zeolite structure and the method of its release, has been proved.

## 1. Introduction

One of the main problems in road construction is high production temperatures and the incorporation of mix asphalt into the road surface. Production of hot mix asphalt (HMA) is associated with high emissions of harmful compounds, fumes and aerosols into the atmosphere. The alternate solution to HMA is warm-mix asphalt (WMA). The production temperature of these mixes is lower by 20–40 °C, which brings many environmental, economic and technological benefits [1,2,3,4]. Warm-mix asphalt technologies have been known and used in the world for many years. Currently, WMA is mainly used in the United States, where it accounts for over 30% of all produced mixes [5]. In other countries, the industrial application of WMA technology is at a much lower level. Among European countries, only in France and Sweden, WMA’s share in overall bitumen production exceeded 10% [6]. In most countries, as in Poland, there is no industrial production of this type of mixes. One of the important reasons for the higher use of WMA in the USA than in European countries is the type of asphalt mix plant [3]. In the United States, the counterflow asphalt mix plant is often used [7]. It is easier to foam asphalt with water at these plants. Over 70% of WMA technologies in the United States are production plant foaming. In Europe, the use of batch plants prevails. In this type of plant, it is easier to foam the asphalt using the indirect method—by dosing the so-called water carriers. An example of this type of material are zeolites—minerals from the group of skeletal aluminosilicates with diversified structure in which there are empty spaces in the form of chambers and channels. A characteristic feature of zeolites is the presence of water molecules in their composition which are bound in a specific way. Releasing the so-called “zeolitic water” under the influence of temperature rise allows to induce the effect of foam asphalt reducing the mix-asphalt production temperature. Asphalt foaming in this technology does not happen suddenly, as in the case of using only water. Zeolite water is released gradually over time, which is why the foaming effect continues during the production, transport and incorporation of mix asphalt into the road pavement.

Genetically zeolites are divided into natural and synthetic ones. The most important zeolite minerals forming deposits include clinoptilolite, philipsite, chabasite and mordenite [8]. Synthetic zeolites are obtained from chemical reagents, mineral raw materials or from waste materials e.g., by-products of coal combustion [9]. These materials have a structure with strictly defined parameters, which enables their implementation in various industries [10,11,12]. Most often zeolites produced in the synthesis process are these with structure of: NaA, NaX, NaY, ZSM-5 (Zeolite Socony Mobil-5) types.

The first studies of WMA technology with the addition of zeolites concerned the use of synthetic zeolites of the structure type Linde A (LTA) produced from chemical reagents [13,14,15,16,17]. In recent years, WMA research has been conducted with synthetic zeolites produced from waste materials. Woszuk et al. used zeolite with NaP1 structure type (GIS-gismondite) obtained from fly ash from coal combustion [18,19]. Zhang et al. used a zeolite with NaA structure type (LTA) formed in the synthesis of sewage sludge ash [20]. Very promising results regarding the properties of asphalts and mix asphalts were obtained using natural zeolite clinoptilolite (HEU-heulandyte) [18,21,22,23].

Since zeolite is not an asphalt modifier and acts as a water carrier, the amount of zeolite water that the material releases at mix asphalt production temperatures is important. In “water storing” technologies, the amount of water that enables effective foaming of an asphalt binder is from 2% to 4% [24]. Recent research indicates that the amount and manner of zeolite water releasing is affected by the type of zeolite, its textural properties and crystallochemical character [19]. Theoretically, one can therefore assess the suitability of a given type of zeolite for asphalt foaming based on its physicochemical properties. The final verification should, however, include rheological tests of asphalts and assessment of the physico-mechanical properties of the warm-mix asphalt with the addition of a new type of material [25,26,27,28].

The use of zeolites produced from fly ash has an advantage over other solutions in terms of environment and is part of the policy of sustainable development. This is especially important in countries where coal is dominating source of energy. In Poland, about 0.1 million tons of annual production of fly ash from coal combustion is temporarily stored, whereas landfill sites contain 26.0 million tons of fly ash [29]. Assuming annual mix asphalt production at the level of 19.0 million tonnes [9] and the amount of zeolitic material dosed in amount of 0.5% in relation to the mix asphalt mass [18]—WMA production with zeolites at the level of 1% of total mix asphalts production will reduce fly ash in the heaps by about 950 tons per year.

The aim of this work is to assess the possibility of using zeolites produced from fly ash with various types of structure in foamed asphalt technology based on their physicochemical properties and results of dynamic viscosity tests on asphalt binders.

## 2. Experimental Materials

Zeolites used in this study represent four significantly different framework topologies. The characteristics of zeolitic materials are presented in the Table 1 [30].

The zeolites used in the study were obtained as products of hydrothermal conversion of fly ash with water solution of NaOH, according to the scheme:

$$\mathrm{fly}\;\mathrm{ashe}+x\;\left[\mathrm{mol}\cdot {\mathrm{dm}}^{-3}\right]\;\mathrm{NaOH}\begin{array}{c} \mathrm{time}\\ \to \\ \mathrm{temperature} \end{array}\mathrm{zeolite}+\mathrm{residue}$$

The aluminosilicates present in fly ash dissolve into the reaction mixture and then cluster, nucleate and grow constantly as crystals on the surface of fly ash particles. Zeolitization process of fly ash particles consists of (a) Dissolution of Al and Si content of fly ash into the solution (b) Condensation of Al and Si polymers (dimers, trimers etc.) on the surface of fly ash particle (c) Nucleation of zeolites (d) Zeolite crystal growth. The type of growing crystals depends on the synthesis paramaters such as the concentration of NaOH solution, NaOH/fly ash ratio and temperature of the system and duration [31,32].

The fly ash used as a substrate for the conversion was obtained from the Power Plant Kozienice. According to the chemical composition it belongs to class F (in the ASTM C 618-08 classification) [33].

The zeolites of NaA, NaX, NaP1 and sodalite type were produced using the prototype of technological line in the following conditions (Table 2).

The article is interdisciplinary, therefore it was decided to simplify nomenclature. The phrase “zeolite material” refers to the material in which the type of zeolite dominates and other minerals are present to some small extent.

Two types of asphalt were used in the tests: 35/50 and 100/150 penetration grade.

In Table 3 the basic properties of asphalt binders and their fraction composition are presented.

Asphalt 35/50 is widely used in asphalt mixes incorporated in road pavements in Poland. Asphalt 100/150 is not used for road pavements in countries with moderate climate, due to very low softening temperature and high ruttings susceptibility. The use of this type of bitumen in this research allowed to assess the effect of the zeolite addition on the asphalt foam effect of significantly different penetration grade bitumen. There is a view that the foaming technology is more effective when using the so-called soft asphalts.

## 3. Research Method

### 3.1. Characterization of Zeolites

Chemical analysis was performed with the energy dispersive X-ray fluorescence method (EDXRF) using the Epsilon 3 Panalytical spectrometer (Panalytical, Almelo, The Netherlands) with RTG Rh 9 W, 50 kV, and 1 mA lamp (Panalytical, Almelo, The Netherlands). The analysis ranged from Na to Am. The sample was air-dried.

Laser diffraction method (Mastersizer 3000 with Hydro G dispersion unit with the measuring range of 0.02 μm to 2 mm) was used to measure the particle size distribution (PSD). For PSD determination Mie theory was applied. The parameters that were used were as follows: 1.52 for light refractive index and 0.1 for absorption coefficient. Measurements were carried out with the pump and stirrer speeds of 1750 and 700 rpm, respectively.

In order to define textural parameters and properties (specific surface area, pore volume, etc.) of the zeolites nitrogen adsorption/desorption isotherms were determined at liquid nitrogen temperature (−194.85 °C) using Accelerated Surface Area and Porosimetry System ASAP 2020M (Norcross, Georgia, GA, USA). Braunauer-Emmett-Teller’s multilayer adsorption theory (BET) at p/p_0_ between 0.06 and 0.3 (p, p_0_—equilibrium pressure and saturation pressure of nitrogen) was applied to determine specific surface area of the zeolites. Pore volume (*V*) was determined from the volume of adsorbed nitrogen at pressure p/p_0_ = 0.98 whereas pore diameters (D_p_) were calculated according to the equation D_p_ = 4 V/S_BET_.

Phase composition of samples were determined using X-ray Diffraction method (XRD) (Panalytical, Almelo, The Netherlands) using X’pert PROMPD diffractometer with PW 3050/60 goniometer, Cu tube and graphite monochromator. The analysis was performed with the angle range of 5° to 65° (2*θ*). The method allows to determine interstitial spacing d_hkl_ characteristic for a particular crystal structure on the basis of Bragg’s law.

Water releasing mechanisms (the amount and temperature range of water release) from structure of the zeolites were analyzed using thermal analysis. Differential Thermal Analysis/Thermogravimetric curves (DTA/TG) (NETZSCH-Gerätebau GmbH, Selb, Germany) were recorded using derivatograph STA 449 F3 Jupiter Netzsch between 25 and 800 °C in air with a heating rate of 10 °C/min. Endothermic and exothermic processes taking place in the sample during heating such as dehydration and structure destruction are reflected by DTA curve while TG curve corresponds to the sample mass loss resulting from these phenomena as a function of time.

### 3.2. Dynamic Viscosity

Dynamic viscosity tests were performed using Brookfield’s viscometer (AMETEK Brookfield, Middleboro, MA, USA) according to ASTM D 4402 [34] at two temperatures corresponding to temperatures occurring during the process of pavement construction, i.e.,

- 135 °C—experienced during paving and compaction.
- 160 °C—during production in the asphalt plant.

That are the temperature characteristics for a typical HMA process. Such testing conditions have so far been used in the evaluation of WMA viscosity with the addition of zeolites and mesoporous siliceous materials [3,35]. Considering the actual WMA production conditions, further tests are needed for asphalt viscosities with zeolites at lower temperatures.

The measurements were done for base asphalt and for bitumens with the addition of zeolites samples. The zeolite material was added in amounts of 3%, 5% and 7% in relation to the bitumen mass. Asphalt was heated in the oven (ORLEN Asfalt, Płock, Poland) to the testing temperature and then zeolite material was added into the hot binder and mixed thoroughly for 1 min. Prepared asphalt specimens were conditioned at the testing temperature in the dryer for 5 min. Next, the samples were transferred to Brookfield viscometer (AMETEK Brookfield, Middleboro, MA, USA). When samples were in the instrument, further conditioning was conducted (for 10 min). Then, the first dynamic viscosity measurements were made. Viscosity measurements were taken at the following time intervals: 15, 30, 45 and 60 min, counted from the end of all two-step 15 min conditioning period. The viscosity tests were performed on separate samples at each temperature.

## 4. Results and Discussion

### 4.1. Zeolite Properties

The chemical composition of zeolite materials used in the studies is shown in Table 4.

The main components in the samples tested are SiO_2_ and Al_2_O_3_. Depending on the type of zeolite they are present in amounts from 34.50% to 43.71% and from 19.75% to 33.80% respectively. Important chemical components of the tested materials are also CaO (4.62–16.61%) and Fe_2_O_3_ (5.87–9.95%) and Na_2_O (4.84–7.56%). Other components are present in small amounts.

**Figure 1:**
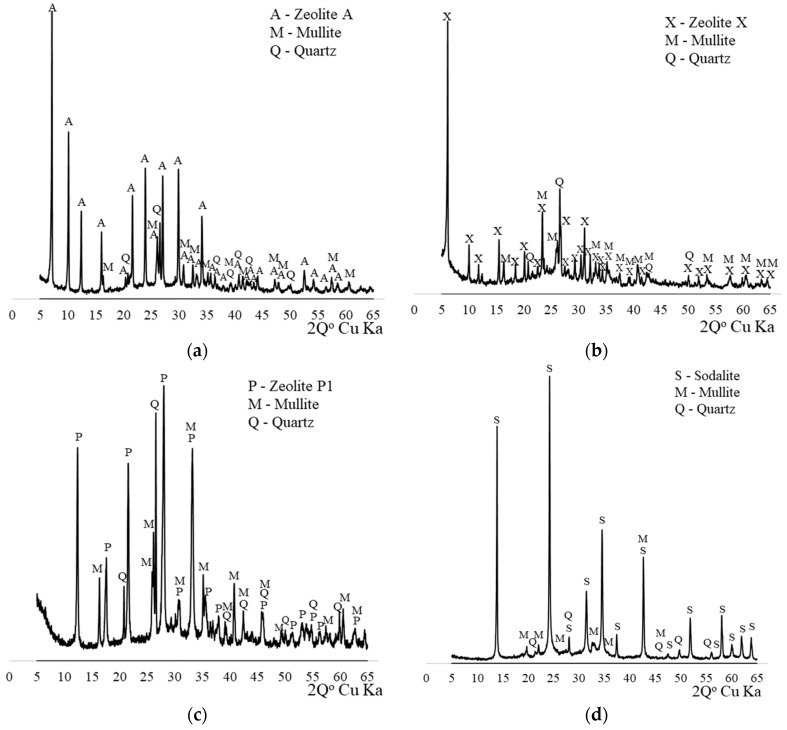
Diffractograms of mineral composition for zeolite materials used in research: (**a**) NaA; (**b**) NaX; (**c**) NaP1; (**d**) sodalite.

The presence of individual zeolite phases in reaction products is confirmed by XRD studies (Figure 1). The quantitative content of zeolites in the material determined by the Rietveld method is respectively: NaA—78%, NaX—82%, NaP1—72% and sodalite 90%. Mullite, quartz and non-converted aluminosilicate glaze were the other components.

**Figure 2:**
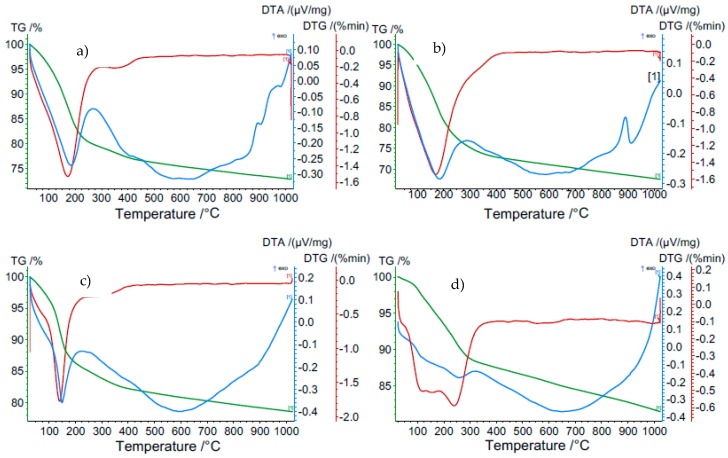
The thermogravimetric analysis (TG) and differential thermal analysis (DTA) graphs zeolite materials used in research: (**a**) NaA; (**b**) NaX; (**c**) NaP1; (**d**) sodalite.

The comparison of thermal curves for the tested zeolite materials is shown in Figure 2.

All registered isotherms for individual zeolites according to the IUPAC (International Union of Pure and Applied Chemistry) are type II isotherms. However, they differ in the size and shape of the hysteresis loop at p/p_0_ > 0.4. For samples NaA, NaX and NaP1, they can be classified as H2/H3 while for sodalite H4 (IUPAC). Such shape is produced by nonuniform pores frequently formed by granular material.

All zeolites show the presence of an intense endothermic peak in the range from 25 °C to 400 °C. This effect is related to the gradual loss of adsorbed and structural water trapped in the zeolite crystals. The nature of this peak and its maximum is strictly dependent on the type of zeolite structure. For NaX, NaA and NaP1 zeolites, this effect is sharp with the maximum for respectively 174 °C, 172 °C and 137 °C. Sodalite has a wide and blurry effect with two maxima at temperatures of 150 °C and 243 °C. These effects are accompanied by a significant decrease in mass on the TG thermal curve. The largest was observed for NaX zeolite—27% and NaA—23%. Slightly lower for NaP1 zeolite—18% and 12% for sodalite. Above 400 °C, there are no exothermic as well as endothermic effects on the thermal curves.

Selected textural parameters of zeolite materials are presented in Table 5.

The tested zeolite material is characterized by a large variety of textural parameters. The largest BET surface area has NaX zeolite—218 m^2^/g, the smallest NaA zeolite—21 m^2^/g. The microporous nature of these zeolites is emphasized by the surface area and micropore volume in the structure of materials used in the study. The micropores are responsible for higher than 70% of the total pore area in zeolites used. Zeolite NaP1 and sodalite, however, have a mesoporous character. The area of mesopores for NaP1 and sodalite is above 90% of the total pore surface (for NaP1 is 90% and for sodalite 93%).

### 4.2. Dynamic Viscosity of Asphalt

**Figure 3:**
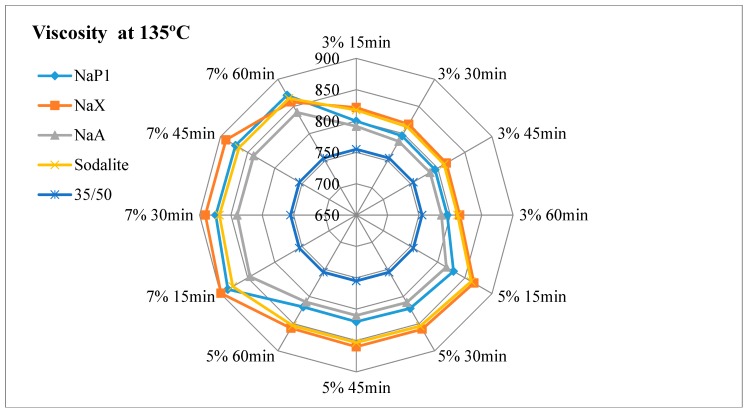
The results of dynamic viscosity tests for 35/50 asphalt with the addition of zeolites measured at 135 °C.

**Figure 4:**
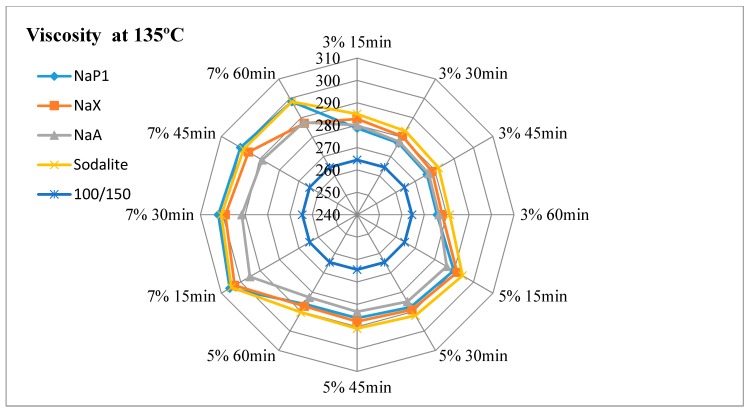
The results of dynamic viscosity tests for 100/150 asphalt with the addition of zeolites measured at 135 °C.

**Figure 5:**
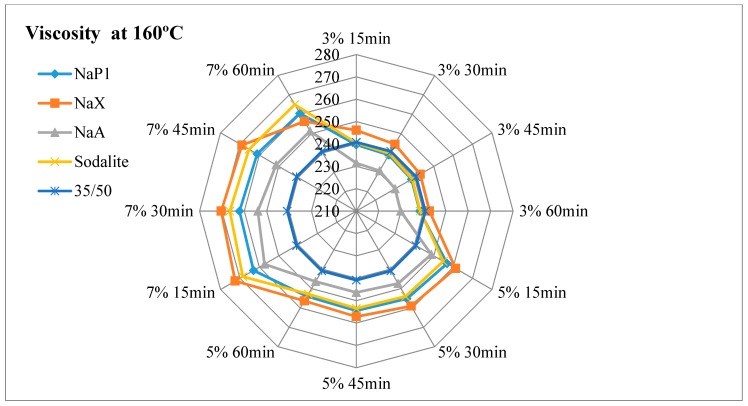
The results of dynamic viscosity tests for 35/50 asphalt with the addition of zeolites measured at 160 °C.

**Figure 6:**
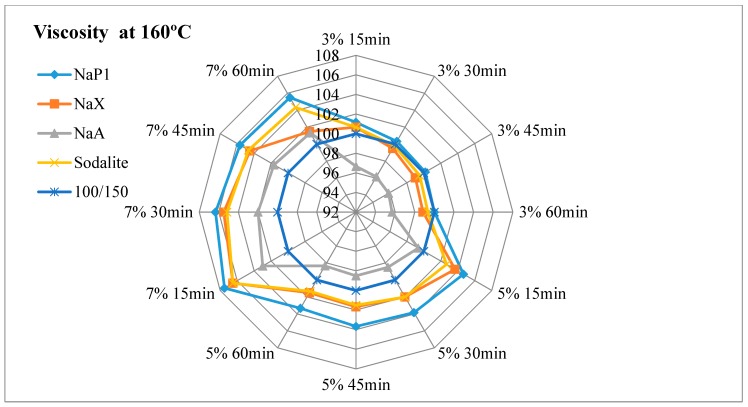
The results of dynamic viscosity tests for 100/150 asphalt with the addition of zeolites measured at 160 °C.

The results of dynamic viscosity tests for bitumens with the addition of zeolites are presented in Figure 3, Figure 4, Figure 5 and Figure 6.

#### 4.2.1. The Effect of the Amount of Zeolite Additive on Asphalt Viscosity

Regardless of the type of zeolitic material used and the test temperature, the asphalt viscosity increased with the increase of zeolite amount added. For samples with asphalt 100/150, this increase was similar to linear and ranged from 1.8% to 4.2%. For mixes with 35/50 bitumen, the increase in viscosity ranged from 1.4% to 7.2%. The organic and chemical additives used in warm-mix asphalt technologies are usually completely soluble and form a homogeneous liquid with asphalt. The zeolite materials are in the form of an insoluble solid. When added to the bitumen, they remain in the form of scattered dust, which results in an increase in asphalt viscosity. The zeolites are not used as an asphalt modifier but only as a “water carrier”. During the production mix asphalt zeolites are added to the mineral mix, and then heated hot asphalt is dosed. Therefore, it is very difficult to determine the optimal addition of zeolitic material only on the basis of viscosity tests. Sengoz with co-workers based on the decrease in asphalt viscosity determined that the optimal zeolites concentration is 5% in relation to the mass of the binder [21,22]. These studies concerned synthetic zeolite from Linde A chemical reagents (Aspha-Min) and natural clinoptilolite. Aspects of asphalt viscosity with zeolites were also dealt with by Akisetty and co-workers. A 6% addition of Aspha-Min zeolite caused an increase in bitumen viscosity [36,37]. The addition of 4%, 6%, and 8% of another zeolite material of the type Linde A (Advera) also resulted in the increase of asphalt viscosity [38].

#### 4.2.2. Influence of Test Time on Asphalt Viscosity

The obtained test results indicate a decrease in viscosity over time for each asphalt-zeolite combination-additive amount-test temperature. The highest level of changes occurred after 30 min of the test (45 min after the mixing of asphalt with zeolite) and ranged from 0.3% to 1.6%. After 45 min of the test, the viscosity level stabilized or decreased from 0.0% to 1.0%. The determination of the viscosity after 60 min indicated further stabilization or change in the range from 0.0% to 0.6%. The obtained results coincide with the analysis of asphalts with the addition of clinoptilolite, NaP1 zeolite and zeolites soaked with water [19]. The decrease in asphalt viscosity over time is caused by the slow release of zeolite water. The nature of zeolite water release affects the possibility of lowering the WMA technological temperatures. The mix asphalt compactibility with zeolite tests indicate that the process of zeolite water releasing can last up to 2 h. The improvement of compactness was followed by after one hour of batch conditioning at compaction temperature [39].

#### 4.2.3. Influence of Test Temperature on Asphalt Viscosity

At 160 °C, at the lowest concentration of zeolite materials, determined viscosity was at or nearby a level of the base asphalt (without the addition of zeolite). The viscosity determined at 135 °C was higher than the results of the basic tests of bituminous binders, regardless of the type of zeolite material and its concentration. The addition of zeolite NaX, NaA and sodalite had a similar effect on the viscosity result at both test temperatures. The use of NaP1 zeolite caused a better foaming effect at 135 °C than at 160 °C. This is related to the releasing manner of zeolite water. Zeolite NaP1 is characterized by a sharp endothermic peak at a temperature of approximately 140 °C, which indicates the loss of a large amount of zeolite water. NaX and NaA Zeolites release the most zeolite water at a temperature of about 180 °C. Sodalite releases zeolitic water in a continuous manner, as evidenced by the lack of clear endothermic peaks in DTA. At temperatures of up to 180 °C zeolites NaX, NaA and NaP1 release a similar amount of zeolite water ranging from 13% to 16%. The smallest amount of water is released by sodalite (approximately 6%).

Based on the above analyzes, it can be concluded that to assess the suitability of the zeolitic material in the warm-mix asphalt technology, not only the amount of zeolitic water is important, but also the way of its release.

#### 4.2.4. The Influence of Properties of the Zeolite Material on Asphalt Viscosity

The lowest viscosity level was obtained for asphalts with the addition of NaA zeolite. This material at temperatures up to 180 °C releases a lot of zeolitic water (about 15%). At the same time it is characterized by a smallest specific surface area (21 m^2^/g) and mesopore surface area (5 m^2^/g). The textural parameters have no influence on the effect of asphalt foaming, but they determine the sorption properties of zeolite materials. The smaller the specific surface area and the porosity, the smaller the sorption. This phenomenon also applies to adsorption of asphalt.

Zeolite NaX is characterized by the highest content of zeolite water—32%. At temperatures up to 180 °C, it releases about 16% of this water. At the same time, the NaX material has the largest specific surface area (218 m^2^/g) and a mesopore surface area (61 m^2^/g). Hence, asphalts with NaX zeolite were characterized by high viscosity. In the case of 35/50 asphalt mixes, the highest results were observed among all tested samples. Even so, porous materials can be used in WMA technology. There is a known way to improve the effect of asphalt foam by adding materials soaked with water [19,35]. One should assume that the NaX zeolite, due to its textural properties, adsorbs a large amount of water. The use of zeolites soaked with water results in a smaller amount of zeolite additive in WMA technology and reduces the production costs of mix asphalt.

The zeolite water content in sodalite is 14.5%. At a temperature of up to 180 °C, about 6% is released, which may be insufficient to cause a foaming effect. This material is also characterized by the low textural parameters (specific surface area 33 m^2^/g, mesopore surface area 31 m^2^/g), hence low adsorption of asphalt. As a result, the viscosity tests results of mixes with sodalite were similar to those obtained on samples with NaX zeolite.

NaP1 zeolite is a material with intermediate specific surface area in relation to the NaX and NaA zeolites. Therefore, the viscosity of asphalt leavens with NaP1 zeolite usually reached intermediate values. The usefulness of this type of zeolite in WMA technology determines the release of zeolite water. It is the only one of the 4 zeolites used in the experiment, characterized by a sharp endothermic peak at 140 °C.

## 5. Conclusions

In recent years, research conducted on this matter indicates the possibility of using zeolite additives in WMA technology. The first works in this regard concerned synthetic zeolite produced from chemical reagents with the structural code LTA. This zeolite contained 21% zeolite water. In many works related to WMA, a different type of zeolite material was used, assuming that the physicochemical properties are unchanged.

The research presented in the paper concerned the evaluation of the possibility of using synthetic zeolites produced from fly ash with 4 different structural types in the WMA production. This technology is part of the sustainable development policy: It reduces the temperature of mix asphalt production through the use of waste materials generated after burning coal in power plants and thermal power plants.

Conducted thermal analyses showed that depending on the zeolite type variable amounts of zeolite water and its release mechanisms are observed. Significant differences also occur in the characteristics of textural parameters, especially the specific surface area and mesopore volume.

The course of the bitumen foaming process by adding zeolites is significantly different from asphalt foaming with water. Zeolites are added to the aggregate mix before the asphalt dosing. This enables the production phases to be kept as for traditional HMAs. It is known that the foaming of asphalt is caused by zeolitic water which is gradually released from the structure of these minerals. The conducted research showed no correlation between the viscosity of asphalt binders and the amount of zeolite water and the way of its release. The textural properties of zeolites, which are not important for the proper asphalt foaming process, have had a major influence on the viscosity of bitumens with the addition of zeolites. As shown in previous studies, the surface area, area and volume of mesopores is important when using zeolites soaked in water [19,35].

When using zeolite materials without modification, the amount of zeolite water contained in the structure of the material and the nature of its release are more important. In the conducted studies, it was confirmed that zeolite materials for which the endothermic peak is present at lower temperatures, allow asphalt to foam at lower temperatures (NaP1 zeolite). It has been shown that synthetic zeolites produced from fly ash can be used in WMA technology. The possibility of asphalt foaming by this type of materials can be concluded on the basis of thermal analyses. When using zeolites without soaking water, the bitumen viscosity testing is of secondary importance. It allows to estimate the time needed to trigger the effect of foaming asphalts by the released zeolitic water. However, it is not possible to determine the optimal amount of zeolite and technological WMA temperatures.

The use of other types of zeolite materials (Linde-A, clinoptilolite) allowed to reduce the production and compaction temperature of the mix asphalt by 20–30 °C.

WMA technology with the addition of synthetic zeolites produced from fly ash requires further research into the assessment of the physical-mechanical properties of mix asphalts.

## Figures and Tables

**Table 1:** Zeolite Framework Types appearing in the zeolite materials used in the research.

Framework Type	Chemical Composition	Channel System	Composite Building Units
NaA (LTA)	Na_96_[Si_96_Al_96_O_384_]·216H_2_O	3-dimensional channel system Channel size: 4.1 × 4.1 Å	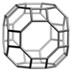
NaX (FAU)	Na_2_[Al_2_Si_2_O_9_] 6H_2_O	3-dimensional channel system Channel size: 7.4 × 7.4 Å	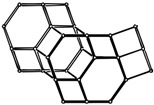
NaP1 (GIS)	Na_6_[Si_10_Al_6_O_32_]·12H_2_O	3-dimensional channel system Channel size: 4.5 × 3.1 Å, 4.8 × 2.8 Å	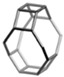
Sodalite (SOD)	Na_8_[Al_6_Si_6_O_24_]Cl_2_	3-dimensional channel system Channel size: 2.2 × 2.2 Å	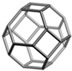

**Table 2:** Synthesis parameters of zeolites materials used in research.

Zeolite Materials	Substrates				Synthesis Conditions	
	Fly Ash (kg)	NaOH (kg)	Aluminum Dust (kg)	H_2_O (dm^3^)	Temperature (°C)	Time (h)
NaA	15	10	0.2	90	90	16
NaX	10	3	-	25	80	48
NaP1	20	12	-	90	80	36
Sodalit	20	18	-	90	95	24

**Table 3:** Properties of the base bitumen.

Test		Specification	Result	
			Asphalt 35/50	Asphalt 100/150
Penetration (25 °C; 0.1 mm)		European standard EN 1426:2009	38.2	100.8
Viscosity at (135 °C) (mPa s)		ASTM D 4402	755	265
Viscosity at (160 °C) (mPa s)			241	100
Fraction composition	Saturate (%)	TLC-FID-thin-layer chromatography with flame-ionization detection	6.5	8.9
	Aromatic (%)		41.1	43.2
	Resin (%)		24.3	29.5
	Asphaltene (%)		28.1	18.3

**Table 4:** Analyses of the chemical compositions of the samples with their main zeolite components given in the header.

Component	NaA	NaX	NaP1	Sodalite
	(% wt)			
SiO_2_	41.20	34.50	43.71	39.90
Al_2_O_3_	29.00	19.75	21.69	33.80
CaO	6.78	11.69	16.61	4.62
MgO	0.55	0.47	1.32	0.75
Fe_2_O_3_	8.67	6.62	5.87	9.95
TiO_2_	0.64	1.63	1.03	0.89
Na_2_O	6.73	6.23	7.56	4.84
K_2_O	1.92	0.54	1.15	0.28
LOI	16.89	15.61	14.37	5.14

**Table 5:** Textural parameters of zeolite materials used in research.

Textural Parameters	NaA	NaX	NaP1	Sodalite
Specific surface area (m^2^/g)	21	218	98	33
Surface of microporous (m^2^/g)	15	156	12	5
Volume of microporous (cm^3^/g)	0.02	0.64	0.01	0.02
Surface of mesoporous (m^2^/g)	5	61	88	31
Volume of mesoporous (cm^3^/g)	0.22	0.14	0.29	0.10
